# N-Acetylneuraminate Pyruvate Lyase Promotes Cell Adaptation to Glucose Deprivation by Regulating Intracellular ATP Levels

**DOI:** 10.3390/cimb48060569

**Published:** 2026-05-29

**Authors:** Zhijun Fan, Yi Li, Shuting Geng, Yu Si, Yuerong Yang, Huali Yu, Xue Hu, Jianhai Jiang

**Affiliations:** NHC Key Laboratory of Glycoconjuates Research, Department of Biochemistry and Molecular Biology, School of Basic Medical Sciences, Fudan University, Shanghai 200032, China; 23211010012@m.fudan.edu.cn (Z.F.);

**Keywords:** hepatocellular carcinoma, N-acetylneuraminate pyruvate lyase, glucose deprivation, energy homeostasis, molecular docking

## Abstract

N-acetylneuraminate pyruvate lyase (NPL) is a key enzyme in sialic acid catabolism that links sialylation to cellular metabolism, but its role in cancer cell metabolic adaptation is poorly defined. In particular, it remains unclear whether NPL contributes to ATP maintenance in hepatocellular carcinoma (HCC) under nutrient stress through its role in sialic acid catabolism. HCC is a highly lethal malignancy characterized by extensive metabolic reprogramming. Here, we investigated whether NPL links sialic acid catabolism to ATP maintenance in HCC cells under glucose deprivation. Glucose deprivation induced NPL expression in Huh7 and PLC/PRF/5 cells, which is consistent with an adaptive response to energetic stress. Stable NPL knockdown reduced intracellular ATP levels. Consistently, cell growth was significantly reduced, as assessed by Cell Counting Kit-8 (CCK-8) and colony formation assays. These effects were more pronounced in the absence of glucose. Exogenous pyruvate partially restored ATP levels and the growth inhibition caused by NPL knockdown, particularly in the absence of glucose. This rescue further suggests that NPL may support ATP maintenance under glucose deprivation partly through pyruvate metabolism. Together, these findings indicate that NPL contributes to maintaining intracellular ATP levels during glucose deprivation, thereby supporting HCC cell adaptation to metabolic stress. To extend these biological findings to potential therapeutic exploration, we performed virtual screening and molecular docking using a U.S. Food and Drug Administration (FDA)-approved drug library. Candidate compounds predicted to bind NPL were identified, providing a basis for further validation and optimization.

## 1. Introduction

Hepatocellular carcinoma (HCC) is the most common type of primary liver cancer and a major cause of cancer mortality worldwide [1]. To date, effective systemic therapeutic options for HCC remain limited. Further studies are needed to identify novel molecular pathways and therapeutic targets involved in tumor progression [2,3,4].

ATP is essential for tumor cells to sustain rapid proliferation and adapt to metabolic stress. Cancer metabolic reprogramming helps sustain ATP supply under nutrient limitation and therapeutic stress while preserving biosynthesis and redox balance [5]. Recent studies have also shown that mitochondrial energy production is not completely impaired in many cancers. These observations provide opportunities to target energy supply during tumor progression and therapy resistance [6]. Therefore, targeting oxidative phosphorylation or substrate availability is emerging as a potential therapeutic strategy [5,7]. In this context, pyruvate is a key intermediate connecting glycolysis and mitochondrial ATP production [8,9]. It can maintain glycolysis in the cytoplasm or enter mitochondria to promote oxidative metabolism, thus affecting cellular energy homeostasis [8,9,10].

While central carbon metabolism plays an important role in tumor energy supply, other metabolic pathways may also contribute to cancer cell adaptation to nutrient stress. Glycosylation pathways are increasingly recognized as regulators of tumor cell behavior and metabolic adaptation [11,12]. Altered sialylation is a common glycosylation change in cancer and has been implicated in tumor progression [13,14]. Together, these observations suggest that sialic acid metabolism may provide a relevant entry point for exploring the connection between tumor sialylation and energy metabolism [15].

N-acetylneuraminate pyruvate lyase (NPL) is a key enzyme in sialic acid catabolism that catalyzes the reversible conversion of N-acetylneuraminic acid (sialic acid) into pyruvate and N-acetyl-D-mannosamine (ManNAc) [15,16]. NPL is highly expressed in the human liver and several other tissues [17]. Recent studies indicate that NPL not only modulates glycoprotein sialylation but may also regulate cellular energy metabolism. In mice with NPL deletion, free sialic acid levels and glycolytic activity are markedly increased, whereas mitochondrial function is partially impaired. These findings suggest a possible link between NPL-dependent changes in sialylation and mitochondrial energy metabolism, with altered sialylation of the mitochondrial leucine-rich pentatricopeptide repeat motif-containing protein (LRP130) implicated as one possible mechanism [15]. Structural studies of recombinant human NPL provide a framework for understanding its enzymatic activity and for exploring potential NPL-targeting compounds [18]. However, it is unclear how NPL affects the growth of HCC cells and whether ATP homeostasis may contribute to this process.

Here, we hypothesized that NPL may contribute to ATP maintenance and proliferative adaptation in HCC cells, particularly under glucose deprivation. To evaluate this hypothesis, we assessed the effects of NPL knockdown on cellular ATP levels and tumor cell growth. We also performed virtual screening and molecular docking to explore candidate small molecules targeting NPL. By clarifying the functional relevance of NPL in HCC cell adaptation to glucose deprivation, this study may provide a basis for future biomarker development and therapeutic exploration.

## 2. Materials and Methods

### 2.1. Cell Culture

Huh7 cells and PLC/PRF/5 cells were obtained from the American Type Culture Collection (ATCC, Rockville, MD, USA). HEK 293T cells were obtained from the National Collection of Authenticated Cell Cultures, Chinese Academy of Sciences (Shanghai, China).

HEK 293T cells were cultured in DMEM supplemented with 10% fetal bovine serum (FBS) and 1% penicillin-streptomycin (P.S.; all from Gibco, Waltham, MA, USA).

During nutrient deprivation, Huh7 and PLC/PRF/5 cells were cultured in DMEM (10% FBS, 1% P.S.; Gibco, Waltham, MA, USA) for 16 h. The culture medium was then replaced with DMEM (BasalMedia, Shanghai, China) containing 25 mM glucose (Sigma-Aldrich, St. Louis, MO, USA), glucose-free DMEM (BasalMedia, Shanghai, China) or amino acid-free DMEM (BasalMedia, Shanghai, China) for 48 h.

For time-course analysis, Huh7 cells were cultured in DMEM containing 25 mM glucose or glucose-free DMEM and harvested at 0 h, 24 h, 48 h and 72 h after medium replacement. The 25 mM glucose condition was used as a standard DMEM culture condition. Glucose-free DMEM was used to induce severe glucose deprivation stress. This condition was used to partially model the limited nutrient supply that tumor cells may encounter in poorly perfused tumor regions [19].

### 2.2. RT-PCR Analysis of NPL Expression

Cells were lysed using TRIzol (Invitrogen, Waltham, MA, USA). Extracted total RNA was used as a template for cDNA synthesis with the RNA PCR kit (Takara Bio, Kusatsu, Japan). Human *NPL* was amplified as the target gene. The specific primers were as follows: forward 5′-CAGGTGATAATTCACGTAGGAGC-3′ and reverse 5′-AACGGTGCAATGACAGCGA-3′. *GAPDH* was used as an internal control for normalization. The PCR reaction mixture was prepared according to the instructions for 2× Phanta Max Master Mix (Vazyme, Nanjing, China). The amplification protocol was set to 30 cycles. Each PCR product was separated on a 1.5% agarose gel in 1× Tris-acetate-EDTA (TAE) buffer and stained with an alternative nucleic acid dye. The bands were visualized and recorded using a UV imaging system (Tanon, Shanghai, China).

### 2.3. Western Blot

Cell lysates were resolved by SDS-PAGE, and the separated proteins were transferred to polyvinylidene difluoride (PVDF) membranes (Roche, Basel, Switzerland). Blocking was performed for 2 h in phosphate-buffered saline with Tween-20 (PBST) containing 5% nonfat dry milk (Sangon Biotech, Shanghai, China). Primary antibodies were diluted at 1:1000 and incubated at 4 °C for 12–16 h, including rabbit polyclonal Anti-NPL antibody (Abmart, Shanghai, China, cat. no. PS11649S) and β-actin monoclonal antibody (Proteintech, Wuhan, China, cat. no. 66009-1-Ig). After washing with PBST, the membranes were incubated with secondary antibody diluted at 1:2000 at room temperature for 1.5–2 h. The membranes were then washed with PBST, followed by the addition of a chemiluminescent substrate and incubation for 2 min before imaging (Tanon, Shanghai, China). Band intensities were quantified using ImageJ software (version 1.33u; National Institutes of Health, Bethesda, MD, USA), and the data were used for statistical analysis.

### 2.4. Plasmid Construction

To achieve stable knockdown of *NPL*, short hairpin RNAs (shRNAs) targeting human *NPL* mRNA were designed and synthesized: shNPL-1: GAGTTTACTGATAGTGCTGAA; shNPL-2: GCTGAAATTCAGTGATACAGA. The shRNA oligonucleotides were heated to 98 °C for 10 min and then allowed to cool gradually to room temperature for annealing. The annealed shRNA oligonucleotides were inserted into the AgeI and EcoRI (New England Biolabs, Ipswich, MA, USA) sites of the pLKO.1-puro vector. The recombinant plasmids were transformed into *E. coli* DH5α. The insert region was verified by sequencing to confirm positive clones.

### 2.5. Lentivirus Production and Infection

A pLKO.1 control plasmid or an NPL shRNA plasmid was co-transfected with the packaging plasmids psPAX2 and pMD2.G into HEK 293T cells using the calcium phosphate method. Viral supernatants were collected 48–72 h later, followed by filtration and concentration. Huh7 and PLC/PRF/5 cells were transduced with lentivirus in the presence of polybrene (Sigma-Aldrich, St. Louis, MO, USA; 8 μg/mL) for more than 72 h to generate stable shNC, shNPL-1, and shNPL-2 cell lines.

### 2.6. RT-qPCR

Total RNA was extracted from shNC and shNPL Huh7 cells using TRIzol (Invitrogen, Waltham, MA, USA). cDNA was synthesized from total RNA using a reverse transcription kit (Takara Bio, Kusatsu, Japan).

Equal amounts of cDNA were mixed with Power SYBR Green PCR Master Mix (Applied Biosystems, Foster City, CA, USA) and primers. Experiments were performed using the ABI Prism 7500 System (Applied Biosystems, Foster City, CA, USA). Data acquisition and preliminary analysis were performed using ABI 7500 Software v2.3 (Applied Biosystems, Foster City, CA, USA). *GAPDH* was used as an internal control for normalization. The relative mRNA expression of *NPL* was calculated using the comparative Ct method to evaluate *NPL* knockdown efficiency in Huh7 cells [20].

### 2.7. Cell Counting Kit-8 (CCK-8) Assay

Cells were seeded into 96-well plates at a density of 3000 cells per well and cultured for 24 h. The medium was then replaced with DMEM containing 25 mM glucose (Sigma-Aldrich), 5.5 mM glucose, or no glucose for subsequent culture. The 25 mM glucose medium was used as the standard culture condition. The 5.5 mM glucose medium was included to approximate physiological glucose availability, while medium without glucose was used as an in vitro model of complete glucose withdrawal [19,21]. Cell growth was assessed at 0 h, 24 h, 48 h, 72 h and 96 h. At each time point, 90 μL of the corresponding culture medium and 10 μL of CCK-8 reagent (Dojindo, Shanghai, China) were added to each well. The plates were incubated at 37 °C for 1 h, and absorbance at 450 nm was measured using a microplate reader (PerkinElmer, Pontyclun, UK).

### 2.8. Colony Formation Assay

Cells were seeded into 6-well plates at 1500 cells per well and cultured for 9–12 days. Cells were fixed with methanol for 20 min and stained with crystal violet at room temperature for 30 min. Plates were washed with phosphate-buffered saline (PBS) to remove excess dye, air dried at room temperature, imaged, and quantified using ImageJ (version 1.33u) [22].

### 2.9. Intracellular ATP Measurement

ATP levels were measured using an ATP assay kit (Beyotime Biotechnology, Shanghai, China). Cells were lysed with ATP lysis buffer for ATP measurement. Matched cell samples were lysed with radioimmunoprecipitation assay (RIPA) buffer for protein normalization (Beyotime Biotechnology, Shanghai, China). ATP detection reagent was added at the recommended ratio according to the instructions. After incubation for 3 min in the dark, cell lysates were added to each well, and chemiluminescence was immediately measured using a multimode microplate reader (PerkinElmer, Pontyclun, UK). Three independently cultured wells were included for each group to account for sample variation. Total protein in the matched cell samples was measured using a bicinchoninic acid (BCA) assay (Beyotime, Shanghai, China). Intracellular ATP levels were normalized to the corresponding total protein content and expressed as ATP per mg protein for comparisons among groups.

### 2.10. Pyruvate Supplementation Assays for Cell Growth and Intracellular ATP

ShNC and shNPL cells were cultured with 25 mM or 0 mM glucose in DMEM (BasalMedia, Shanghai, China), either without pyruvate or with 1 mM pyruvate supplementation (Beyotime, Shanghai, China). Cell growth was assessed at 12 h, 24 h, and 36 h using the CCK-8 (Dojindo, Shanghai, China) assay as described above. Intracellular ATP levels were measured after 36 h of pyruvate supplementation using the same method described above.

### 2.11. Virtual Screening and Molecular Docking

The structural information of NPL protein was obtained from UniProt (release 2026_01; accession Q9BXD5, https://www.uniprot.org/, accessed on 20 April 2026) [23,24], including links to corresponding Protein Data Bank (PDB) structures. Potential binding pockets were predicted using the Prankweb online server (PrankWeb 4, https://prankweb.cz/, accessed on 20 April 2026) [25]. A library of U.S. Food and Drug Administration (FDA)-approved drugs was downloaded from PubChem (https://pubchem.ncbi.nlm.nih.gov/, accessed on 20 April 2026) [26]. Virtual screening was performed using AutoDock Vina (version 1.1.2; Scripps Research, La Jolla, CA, USA) to identify small molecules predicted to bind to the putative NPL binding pocket [27,28]. The exhaustiveness value was set to the default value of 8, and compounds with predicted binding energies more negative than −4.0 kcal/mol were retained for preliminary screening. This relatively permissive cutoff was selected based on the distribution of predicted binding energies in the initial virtual screening. The value of −4.0 kcal/mol was used to exclude compounds with higher predicted binding energies and to retain those with more negative values. This allowed compounds with possible low or intermediate predicted binding potential for NPL to be included for further evaluation. These compounds were then further evaluated using parameters such as docking scores and consistency of predicted binding poses. The structures of the screened compounds were obtained from the PubChem database and redrawn using Ketcher (version 3.12.0; EPAM, Newtown, PA, USA; https://lifescience.opensource.epam.com/ketcher/, accessed on 20 April 2026) [29].

Interactions between selected compounds and NPL protein were further characterized by molecular docking. Water molecules and the co-crystallized ligand were removed from the target protein structure using PyMOL (version 2.6.0; Schrödinger, New York, NY, USA) [30]. The prepared structure was imported into AutoDockTools (version 1.5.7; Scripps Research, La Jolla, CA, USA) to add polar hydrogens, assign Gasteiger charges, and merge nonpolar hydrogens. The docking grid box was centered at *x* = 9.092, *y* = 74.233, and *z* = 31.096 Å, with 126 × 126 × 126 grid points and a grid spacing of 0.786 Å. Molecular docking was then carried out using these grid parameters [31]. The docking results were visualized using PyMOL [30].

### 2.12. Dataset and Bioinformatics Analyses

In this study, the HCCDB v2.0 database was used to visualize the spatial distribution of *NPL* expression. The UALCAN database was used to analyze differences in *NPL* expression between normal liver and HCC tumor samples. The UALCAN expression analysis included 50 normal liver samples and 371 primary HCC tumor samples. Survival analyses were performed using UALCAN. Patients were divided into a high *NPL* expression group (*n* = 91) and a low or medium *NPL* expression group (*n* = 274) according to the grouping method provided by UALCAN. *p* < 0.05 was considered statistically significant. Plots and summary data were obtained from the publicly accessible HCCDB v2.0 (https://www.lifeome.net/database/hccdb/, accessed on 20 April 2026) [32] and UALCAN (https://ualcan.path.uab.edu/, accessed on 20 April 2026) [33] web portals.

### 2.13. Statistical Analysis

Statistical analyses were conducted with GraphPad software (Prism 10.1.2, GraphPad, Boston, MA, USA). Data are shown as the mean ± SD from three experiments. Differences were analyzed using a two-tailed Student’s *t* test, one-way ANOVA with Tukey’s test or two-way ANOVA with Tukey’s test. Statistical significance was defined by *p* values (* *p*  <  0.05; ** *p*  <  0.01; *** *p*  <  0.001).

## 3. Results

### 3.1. High NPL Expression in HCC Is Associated with Poor Prognosis

**Figure 1:**
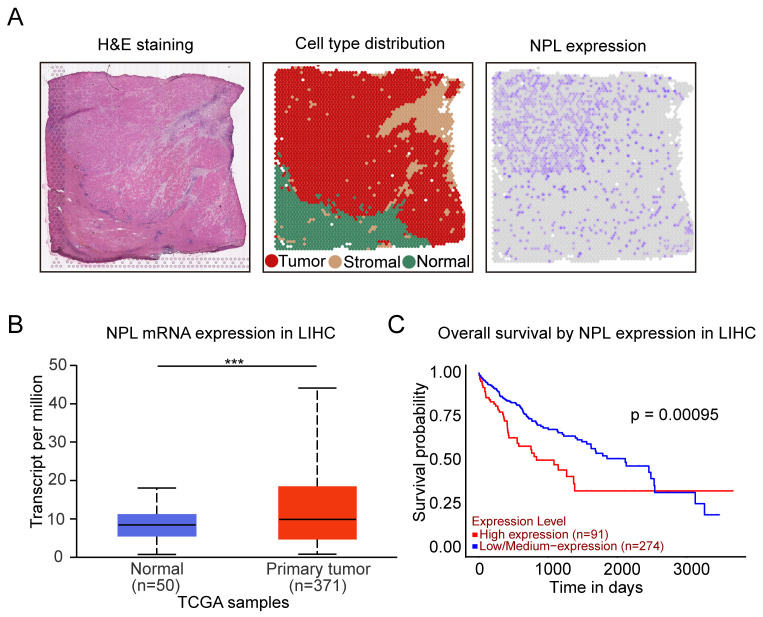
Public database analyses of N-acetylneuraminate pyruvate lyase (NPL) expression and prognostic association in hepatocellular carcinoma (HCC). (**A**) Spatial transcriptomic visualization from a representative HCC patient in HCCDB v2.0 showing cropped areas of H&E staining and cell type distribution, together with the corresponding *NPL* spatial expression. Data source: https://www.lifeome.net/database/hccdb/. (**B**) *NPL* mRNA expression in normal liver and primary HCC tissues from the TCGA-LIHC cohort; median transcripts per million (TPM), primary tumor vs. normal: 9.776 vs. 8.311; *** *p* < 0.001. (**C**) Kaplan–Meier analysis of overall survival in HCC patients based on *NPL* expression in the TCGA-LIHC cohort. Data source: https://ualcan.path.uab.edu/.

Spatial transcriptome analysis of a representative HCC patient from the HCCDB v2.0 database showed relatively higher *NPL* expression in tumor regions than in adjacent normal regions (Figure 1A). *NPL* mRNA expression was significantly higher in primary HCC tissues than in normal liver tissues in the TCGA-LIHC cohort (*p* = 5.30 × 10^−9^; Figure 1B). Kaplan–Meier analysis of the TCGA-LIHC cohort using UALCAN showed that HCC patients with high *NPL* expression had shorter overall survival (log rank *p* = 0.00095; Figure 1C).

Taken together, these public database analyses suggest that *NPL* is upregulated in HCC tissues and that elevated *NPL* expression is associated with poor prognosis in HCC.

### 3.2. NPL Expression Increases Under Glucose Deprivation

**Figure 2:**
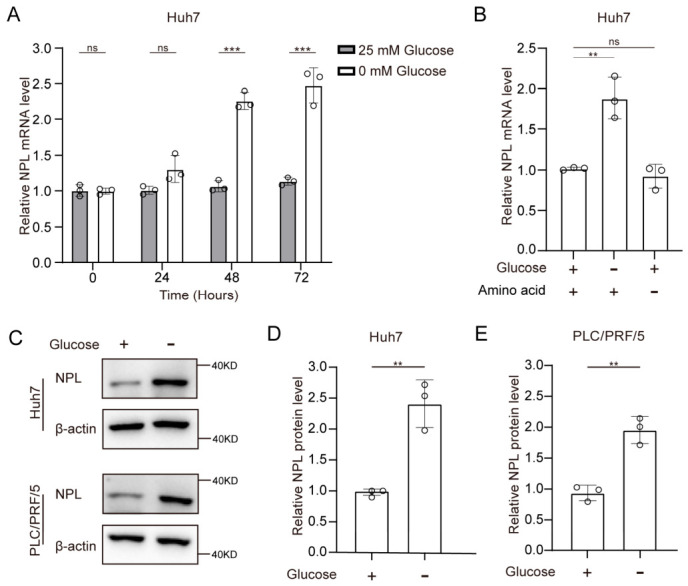
NPL expression under glucose deprivation. (**A**) RT-qPCR analysis of *NPL* mRNA expression in Huh7 cells cultured in 25 mM glucose or 0 mM glucose medium for 0 h, 24 h, 48 h, and 72 h. *NPL* mRNA levels were expressed relative to the 0 h 25 mM glucose group. Data are presented as mean ± SD from three experiments; ns = not significant, *** *p* < 0.001, two-way ANOVA with Tukey’s test. (**B**) RT-PCR analysis of *NPL* mRNA expression in Huh7 cells under nutrient deprivation. Data are presented as mean ± SD from three experiments; ns = not significant, ** *p* < 0.01, one-way ANOVA with Tukey’s test. (**C**) Western blot analysis of NPL protein expression in Huh7 and PLC/PRF/5 cells under glucose deprivation. (**D**,**E**) Quantification of NPL protein expression in Huh7 and PLC/PRF/5 cells. Data are presented as mean ± SD from three experiments; ** *p* < 0.01, two-tailed Student’s *t* test.

During tumor progression, abnormal angiogenesis and insufficient perfusion expose tumors to chronic microenvironmental stress, including hypoxia and glucose deprivation. These conditions have been implicated in tumor growth, therapy resistance, and immune evasion [34,35]. To delineate the impact of nutrient limitation on NPL expression in HCC cells, we examined *NPL* mRNA expression in Huh7 cells under glucose deprivation at 0 h, 24 h, 48 h, and 72 h. Under 25 mM glucose, *NPL* expression remained essentially unchanged across the time course. By contrast, glucose deprivation caused only a modest change in *NPL* mRNA at 24 h, followed by a marked increase at 48 h and 72 h (Figure 2A). Based on this response pattern and the cell status under glucose deprivation, 48 h was used as the main endpoint for subsequent nutrient deprivation experiments.

We then modeled nutrient limitation in the tumor microenvironment by culturing cells in medium without amino acids or glucose. RT-PCR showed that *NPL* mRNA levels in Huh7 cells did not change significantly under amino acid deprivation. In contrast, glucose deprivation significantly increased *NPL* expression compared with the control condition (Figure 2B). This increase was also observed at the protein level when glucose was absent (Figure 2C,D), suggesting that NPL responds to glucose limitation in HCC cells. In parallel, PLC/PRF/5 cells were used for independent validation and showed consistent findings (Figure 2C,E). Together, these results indicate that NPL expression is induced under glucose deprivation in HCC cells.

### 3.3. Stable NPL Knockdown in Huh7 and PLC/PRF/5 Cells

**Figure 3:**
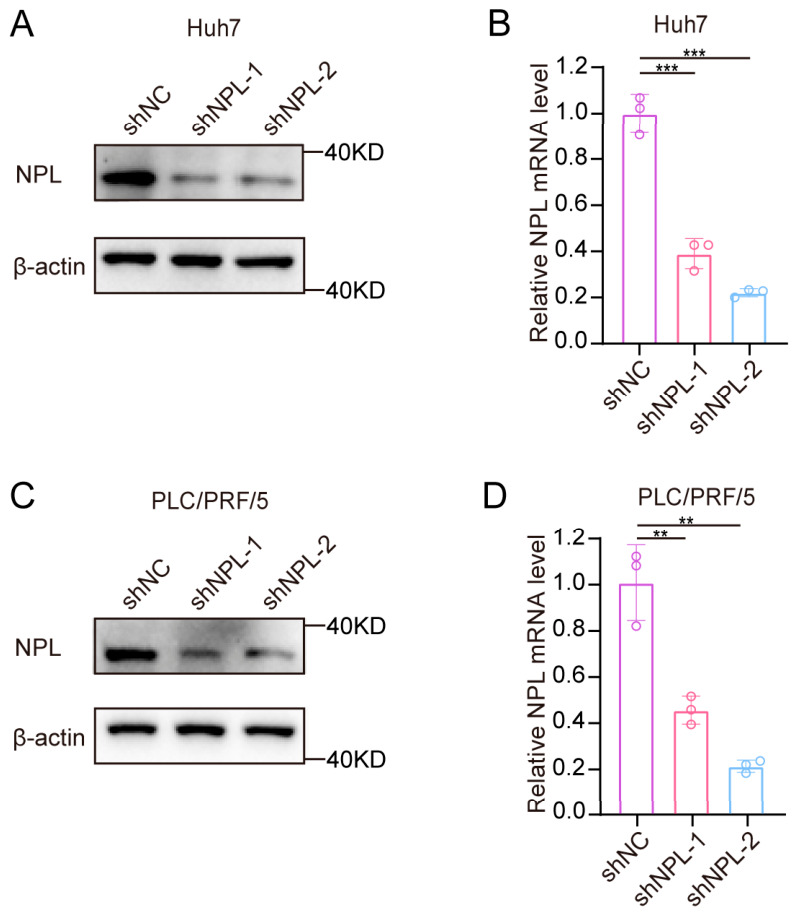
Stable NPL knockdown in Huh7 and PLC/PRF/5 cell lines. (**A**) Western blot analysis of NPL knockdown efficiency in Huh7 cells. (**B**) RT-qPCR analysis of NPL knockdown in Huh7 cells. Data are presented as mean ± SD from three experiments; *** *p* < 0.001, one-way ANOVA with Tukey’s test. (**C**) Western blot analysis of NPL knockdown efficiency in PLC/PRF/5 cells. (**D**) RT-qPCR analysis of NPL knockdown in PLC/PRF/5 cells. Data are presented as mean ± SD from three experiments; ** *p* < 0.01, one-way ANOVA with Tukey’s test.

Stable Huh7 and PLC/PRF/5 cell lines expressing shRNAs targeting *NPL* were generated, and knockdown efficiency was evaluated by Western blotting and RT-qPCR. RT-qPCR analysis showed that *NPL* mRNA levels were reduced by approximately 55% to 78% in shNPL cells compared with shNC cells. Western blotting further confirmed reduced NPL protein expression in both cell lines (Figure 3). These validated knockdown cell lines were then used to examine the functional role of NPL in ATP maintenance and cell growth.

### 3.4. NPL Knockdown Reduces Cell Growth and Colony Formation

**Figure 4:**
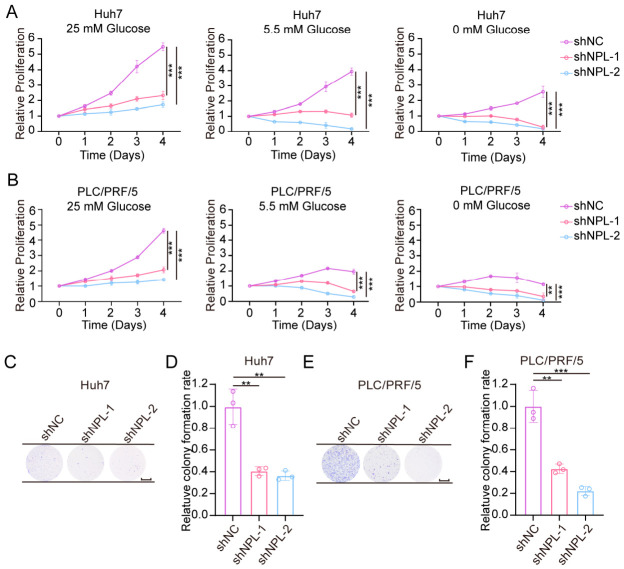
Effects of NPL knockdown on cell growth and colony formation in Huh7 and PLC/PRF/5 cells. (**A**) Cell Counting Kit-8 (CCK-8) assay of Huh7 cell growth; *** *p* < 0.001. (**B**) CCK-8 assay of PLC/PRF/5 cell growth. Data are presented as mean ± SD from three experiments; ** *p* < 0.01, *** *p* < 0.001, two-way ANOVA with Tukey’s test. Values were normalized to the corresponding day 0 value within each glucose condition. (**C**,**D**) Colony formation of Huh7 cells after NPL knockdown. Scale bar = 10 mm, data are presented as mean ± SD from three experiments; ** *p* < 0.01, one-way ANOVA with Tukey’s test. (**E**,**F**) Colony formation of PLC/PRF/5 cells after NPL knockdown. Scale bar = 10 mm, data are presented as mean ± SD from three experiments; ** *p* < 0.01, *** *p* < 0.001, one-way ANOVA with Tukey’s test.

The CCK-8 assay was performed to evaluate cell growth under different glucose concentrations. In medium containing 25 mM glucose, cell growth in shNPL cells was significantly reduced compared with control cells. When cells were cultured in 5.5 mM glucose or glucose-free medium, cell growth was further suppressed in all groups, with a more pronounced reduction in NPL knockdown cells. These findings suggest that NPL knockdown impairs HCC cell growth, particularly when glucose availability is limited (Figure 4A). Consistent results were also observed in PLC/PRF/5 cells (Figure 4B). Further statistical analysis showed a significant interaction between glucose concentration and shRNA group in both Huh7 and PLC/PRF/5 cells, indicating that the effect of NPL knockdown on cell growth varied with glucose availability (*p* < 0.001).

A colony formation assay was performed to evaluate the long term growth capacity of HCC cells following NPL knockdown. Representative images showed that shNPL cells generated visibly fewer and smaller colonies than shNC cells. Quantitative analysis showed a significant reduction in relative colony number in shNPL cells compared with shNC controls (Figure 4C,D). Similar results were observed in PLC/PRF/5 cells (Figure 4E,F).

In summary, these results support a role for NPL in regulating the growth and proliferation of HCC cells.

### 3.5. Knockdown of NPL Reduces Intracellular ATP Levels in HCC Cells

ATP is the primary energy source supporting cellular activities, and pyruvate serves as a key metabolic substrate for ATP production. NPL is a key enzyme in sialic acid catabolism that contributes to pyruvate generation. Since cell growth is closely linked to cellular energy status, we next measured intracellular ATP levels in NPL knockdown cells.

**Figure 5:**
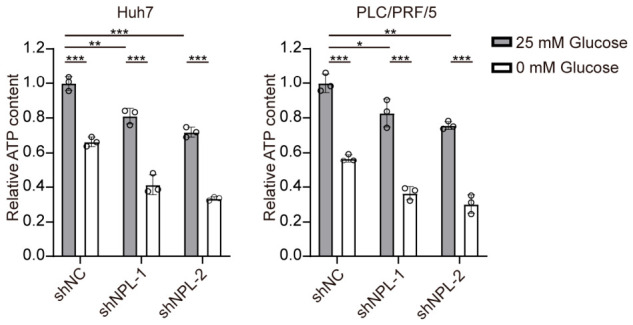
Effects of NPL knockdown on ATP levels in HCC cells. Intracellular ATP levels were measured in Huh7 and PLC/PRF/5 cells cultured in medium containing 25 mM or 0 mM glucose. Data are presented as mean ± SD from three experiments; * *p* < 0.05; ** *p* < 0.01, *** *p* < 0.001, two-way ANOVA with Tukey’s test.

Intracellular ATP was measured in each group under 25 mM glucose and under glucose deprivation. Under 25 mM glucose, ATP levels were significantly lower in shNPL cells than in shNC cells. This reduction was more pronounced under glucose deprivation. Similar results were observed in both Huh7 and PLC/PRF/5 cells (Figure 5). These results were generally consistent with the stronger inhibition of cell growth observed under glucose deprivation.

### 3.6. Pyruvate Supplementation Partially Restores ATP Levels and Cell Growth in HCC Cells

**Figure 6:**
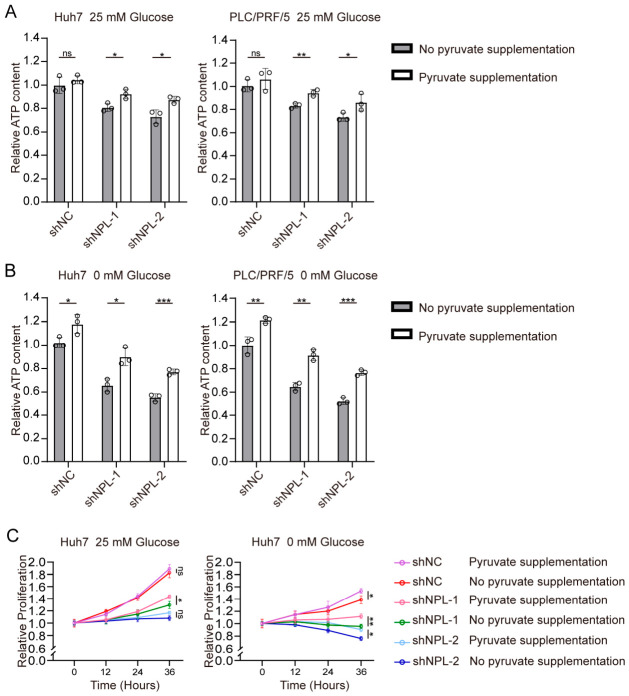
Effects of pyruvate supplementation on ATP levels and cell viability in NPL knockdown cells. (**A**) Intracellular ATP levels of each group with or without 1 mM pyruvate supplementation in medium containing 25 mM glucose. Data are presented as mean ± SD from three experiments; * *p* < 0.05, ** *p* < 0.01, ns = not significant, two-way ANOVA with Tukey’s test. (**B**) Intracellular ATP levels of each group with or without 1 mM pyruvate supplementation in medium containing 0 mM glucose. Data are presented as mean ± SD from three experiments; * *p* < 0.05, ** *p* < 0.01, *** *p* < 0.001, two-way ANOVA with Tukey’s test. (**C**) Cell growth following the addition of 1 mM pyruvate in 25 mM glucose or 0 mM glucose medium, measured by CCK-8. Data are presented as mean ± SD from three experiments; * *p* < 0.05, ** *p* < 0.01, ns = not significant, two-way ANOVA with Tukey’s test. Values were normalized to the corresponding day 0 value within each glucose condition.

We next supplemented the culture medium with exogenous pyruvate to examine whether pyruvate availability could contribute to the ATP and growth phenotypes associated with NPL knockdown. In medium containing 25 mM glucose, pyruvate supplementation did not substantially change ATP levels in control cells but modestly increased them in shNPL cells (Figure 6A). These findings suggest that exogenous pyruvate may partially support ATP maintenance in NPL knockdown cells. When glucose availability was limited, pyruvate supplementation increased ATP levels in all groups, with a more pronounced increase in NPL knockdown cells (Figure 6B). Consistent with these findings, pyruvate supplementation partially rescued the growth defect associated with NPL knockdown under 25 mM glucose. A statistically significant increase was observed in shNPL-1 cells, whereas shNPL-2 cells showed an upward trend but did not reach statistical significance. The rescue effect was more evident under glucose-free conditions (Figure 6C). Together, these results suggest that pyruvate supplementation partially alleviated the ATP and cell growth defects associated with NPL knockdown, particularly under glucose deprivation.

### 3.7. Virtual Screening and Molecular Docking of Small-Molecule Drugs Targeting NPL

**Figure 7:**
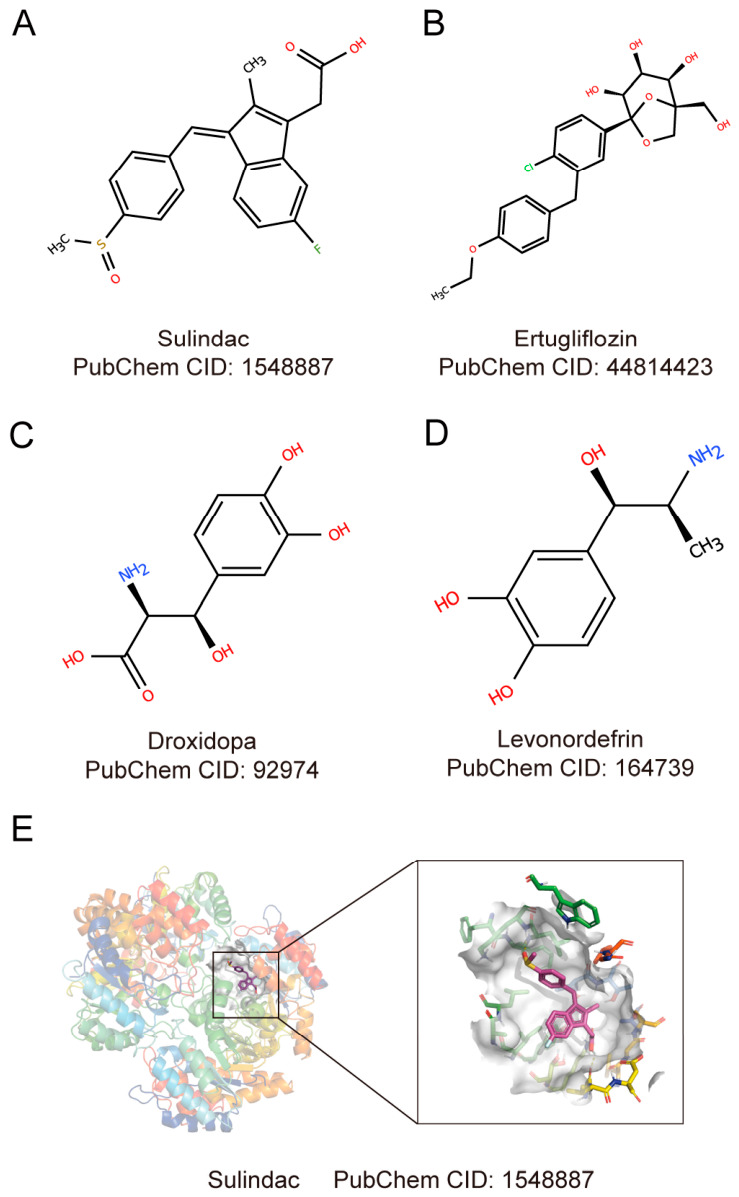
Binding modes of virtually screened small molecules with NPL. (**A**–**D**) 2D chemical structures of four compounds identified by virtual screening and predicted to bind the NPL active site. (**E**) Binding modes of the compound with the lowest predicted binding energy at the NPL active site. The left panels show the overall 3D views of the protein ligand complex, with the protein shown as a cartoon and the ligands shown as sticks within the binding pocket. The right panels provide magnified views of the binding interface; the protein surface is shown in white to illustrate shape complementarity and the ligand (magenta sticks) and key interacting residues are shown as sticks.

Through virtual screening using AutoDock Vina, we identified four compounds predicted to bind the NPL active site pocket: sulindac (PubChem CID: 1548887), ertugliflozin (PubChem CID: 44814423), droxidopa (PubChem CID: 92974), and levonordefrin (PubChem CID: 164739) (Figure 7A–D). To evaluate the robustness of these four compounds, we performed cross-docking and generated 10 docking poses for each compound. Rather than selecting the pose with the lowest predicted binding energy, we assessed pose consistency by clustering all poses.

We observed that several poses occupied a similar region within the predicted NPL binding pocket. Because AutoDock assumes a rigid receptor, these poses did not always correspond to the lowest score. Among the screened compounds, sulindac showed the most favorable binding energy, with a docking score of approximately −10 kcal/mol, and displayed a consistent binding pose (Figure 7E). Sulindac is a nonsteroidal anti-inflammatory drug that acts mainly through cyclooxygenase inhibition. The predicted sulindac pose preserved key interactions with Lys175. The consistency among repeated docking poses increased confidence in this predicted binding mode. These computational results suggest that sulindac may be prioritized as a preliminary candidate for subsequent biochemical evaluation.

## 4. Discussion

To sustain continuous proliferation and adapt to hypoxia and nutrient fluctuations, tumor cells often exhibit metabolic reprogramming centered on ATP supply and energy homeostasis [36,37]. The Warburg effect describes the preferential use of aerobic glycolysis by cancer cells, leading to sustained lactate production even when oxygen is sufficient [36,38]. Evidence suggests that mitochondrial metabolism and oxidative phosphorylation are not universally suppressed in cancer. Instead, they may support metabolic plasticity, stress adaptation, and therapy resistance [39,40,41]. Metabolic reprogramming is also prevalent in HCC [42,43]. Metabolomics studies suggest that HCC exhibits a transition in bioenergetic metabolism from mitochondrial oxidation toward glycolysis, and that the extent of this transition differs across HCC subtypes [44,45,46]. Recent reviews have also highlighted coordinated alterations in glucose, lipid, and amino acid metabolism in HCC [43,47]. Accordingly, focusing on ATP production in tumor cells provides a useful entry point to analyze energetic remodeling in HCC. This helps elucidate how tumor cells survive in complex microenvironments and is important for uncovering relevant metabolic targets [37,48,49]. During sialic acid catabolism, NPL generates pyruvate. Thus, changes in NPL activity may be associated with pyruvate availability and intracellular ATP levels [50,51]. Previous studies further indicate that NPL perturbation is associated with enhanced glycolysis and partial mitochondrial dysfunction [15,52].

Consistent with this metabolic background, our analysis of publicly available HCC datasets showed that NPL expression was altered in HCC tissues and was associated with patient prognosis. These findings based on public databases should be interpreted as associative because public cohorts may differ in sample composition and analytical platforms. Independent cohort validation is still needed to further support the clinical relevance of NPL in HCC.

In the present in vitro model, glucose deprivation induced NPL upregulation in both Huh7 and PLC/PRF/5 cells, suggesting that NPL expression is responsive to glucose deprivation in HCC cells. However, this response may also be influenced by medium composition and other nutrient or microenvironmental factors. The consistent response observed in both cell lines strengthens this observation, although whether it extends to additional HCC cell lines, normal hepatocytes, or other cancer models remains to be determined. Amino acid availability and glutamine metabolism are relevant to this question because amino acid metabolism is closely connected with energy supply, redox balance, and stress responses [53]. In our nutrient deprivation experiment, amino acid deprivation did not induce a comparable increase in NPL expression, suggesting that NPL upregulation may be more closely associated with glucose deprivation than with nutrient deprivation in general. This also indicates that different metabolic stresses may regulate NPL expression through distinct mechanisms. In the tumor microenvironment, hypoxia is another major stress condition in poorly perfused regions and may interact with nutrient limitation to influence metabolic adaptation, including pathways such as glycolysis and mitochondrial metabolism [54]. The specific effects of glutamine deprivation and hypoxia were not examined in the present study. These factors should be examined in future studies to clarify the regulation of NPL under metabolic stress.

NPL knockdown reduced cell growth and colony formation. Combined with the decrease in intracellular ATP and the partial rescue by pyruvate supplementation, these findings are consistent with the possibility that impaired energy supply contributes to the growth suppression associated with NPL knockdown. A possible explanation is that glucose deprivation limits glycolytic ATP generation and pyruvate supply from glycolysis, thereby increasing the relative importance of pyruvate sources independent of glycolysis or compensatory metabolic pathways [15,55,56,57]. The stronger rescue in the absence of glucose suggests that pyruvate availability may become more important when glycolytic input is limited. The incomplete rescue, together with the less evident rescue observed in shNPL-2 cells, also indicates that pyruvate availability is unlikely to be the only mechanism involved [58]. It is also worth noting that only one concentration of exogenous pyruvate was tested, so these data do not define the relationship between pyruvate concentration and ATP or growth rescue. These findings support a possible contribution of pyruvate availability to ATP maintenance when glucose is absent [59]. However, these data do not determine whether NPL knockdown reduces endogenous pyruvate production from sialic acid catabolism. Metabolic flux analysis or isotope tracing will be required to clarify this relationship [60,61,62].

It should also be noted that the magnitude of ATP reduction did not completely parallel the extent of growth inhibition. One possible explanation is that intracellular ATP levels mainly reflect cellular energy status at the time of measurement, whereas cell growth reflects an integrated response to metabolic stress over a longer period. The relationship between cellular ATP levels and cell growth may be influenced by compensatory metabolic responses under different nutrient conditions [63]. This could partly explain the relatively modest ATP decrease after NPL knockdown and the limited growth rescue by pyruvate supplementation under 25 mM glucose conditions. These findings should not be interpreted as evidence that changes in ATP levels directly determine the extent of growth inhibition. Instead, they suggest that ATP maintenance may partly contribute to the regulation of cell growth involving NPL under glucose deprivation. At the cellular level, CCK-8 and colony formation assays do not distinguish reduced proliferation from increased cell death, and apoptosis or cell cycle analyses would be needed to define the cellular basis of this growth phenotype [64]. Although two independent shRNAs were used to reduce concerns that the observed effects were caused by shRNA sequence specificity, genetic rescue experiments would further strengthen the causal link between NPL knockdown and the observed phenotypes.

NPL knockdown may influence the connection between sialic acid catabolism and cellular energy metabolism, which is consistent with the observed decrease in intracellular ATP levels and reduced cell growth [65,66,67]. Intracellular ATP production is closely linked to mitochondrial function and related metabolic processes [68,69]. In the present study, we did not directly examine mitochondrial abundance, morphology, respiration, lactate production, tricarboxylic acid (TCA) cycle intermediates, or metabolic flux assessed by isotope tracing. Whether NPL knockdown alters mitochondrial metabolism or other compensatory pathways remains unclear. Reduced ATP levels may also influence cell proliferation and survival through signaling pathways that respond to ATP levels, which requires further investigation [70,71]. Pyruvate is a key metabolic node that can enter mitochondria to support the TCA cycle and oxidative phosphorylation [72,73,74]. Pyruvate also participates in redox regulation and multiple metabolic pathways, so the rescue effect of exogenous pyruvate should not be interpreted simply as replacement of an NPL derived metabolite [75,76,77].

As a translational extension of the biological findings, we performed virtual screening and molecular docking based on the NPL structure. By predicting ligand conformations and binding preferences within the NPL binding pocket, this approach generated structural hypotheses for candidate compounds. Such analyses can prioritize compounds for further experimental testing and provide structural insights, but they cannot demonstrate direct binding, enzymatic inhibition, or cellular target engagement [78,79]. Although the retained candidate showed stronger predicted binding than the modest threshold used for initial screening, docking scores should be interpreted comparatively and do not establish biological activity [80]. The candidate should therefore be considered a preliminary computational candidate pending biochemical and cellular validation.

Taken together, our data show that glucose deprivation induces NPL expression in HCC cells. NPL knockdown reduced ATP levels and cell growth, both of which were partially rescued by exogenous pyruvate. These findings are consistent with a role for NPL in ATP maintenance under glucose deprivation and suggest a possible contribution of pyruvate metabolism. However, direct metabolic evidence is still needed to determine whether NPL affects endogenous pyruvate production from sialic acid catabolism or related metabolic flux. Future studies using isotope tracing, mitochondrial function assays, genetic rescue, and in vivo models will be needed to define how NPL contributes to metabolic adaptation in HCC. Additional biochemical and cellular validation will also be required to evaluate the candidate identified by docking.

## 5. Conclusions

This study identifies NPL as a potential metabolic node associated with the adaptation of HCC cells to glucose deprivation. When glucose availability was reduced, NPL expression was elevated in multiple HCC cell lines. NPL knockdown suppressed HCC cell growth and was accompanied by decreased intracellular ATP levels. Pyruvate supplementation partly restored ATP levels and cell growth, supporting a possible contribution of NPL to ATP maintenance when glucose availability is limited. Although these findings are limited to in vitro systems, they suggest that NPL may represent a potential metabolic vulnerability worthy of further investigation in HCC. Future studies using in vivo models and experimental validation of the candidate identified by virtual screening may help further evaluate the biological and translational relevance of NPL in HCC.

## Data Availability

The original contributions presented in this study are included in the article. Further inquiries can be directed to the corresponding author. Publicly available data used in this study were obtained from UALCAN and HCCDB v2.0, including data derived from the TCGA-LIHC cohort, all accessed on 20 April 2026.

## Abbreviations

The following abbreviations are used in this manuscript:

HCC	Hepatocellular Carcinoma
NPL	N-acetylneuraminate Pyruvate Lyase
ManNAc	N-acetyl-D-mannosamine
ATCC	American Type Culture Collection
DMEM	Dulbecco’s Modified Eagle Medium
FBS	Fetal Bovine Serum
P.S.	Penicillin Streptomycin
GAPDH	Glyceraldehyde 3-phosphate Dehydrogenase
TAE	Tris–Acetate–EDTA
SDS-PAGE	Sodium Dodecyl Sulfate–Polyacrylamide Gel Electrophoresis
PVDF	Polyvinylidene Difluoride
PBST	Phosphate-Buffered Saline with Tween 20
RT-qPCR	Reverse Transcription Quantitative Polymerase Chain Reaction
CCK-8	Cell Counting Kit-8
PBS	Phosphate Buffered Saline
RIPA	Radioimmunoprecipitation Assay
BCA	Bicinchoninic Acid
PDB	Protein Data Bank
FDA	U.S. Food and Drug Administration
OS	Overall Survival
DFS	Disease-free Survival
LIHC	Liver Hepatocellular Carcinoma
